# Preventing academic difficulties in preterm children: a randomised controlled trial of an adaptive working memory training intervention – IMPRINT study

**DOI:** 10.1186/1471-2431-13-144

**Published:** 2013-09-16

**Authors:** Leona Pascoe, Gehan Roberts, Lex W Doyle, Katherine J Lee, Deanne K Thompson, Marc L Seal, Elisha K Josev, Chiara Nosarti, Susan Gathercole, Peter J Anderson

**Affiliations:** 1 Murdoch Childrens Research Institute, Royal Children’s Hospital, Flemington Road, Parkville, Victoria, Australia; 2 Department of Paediatrics, The University of Melbourne, Royal Children’s Hospital, Flemington Road, Parkville, Victoria, Australia; 3 Clinical Research Development, Royal Women’s Hospital, Grattan Street, Parkville, Victoria, Australia; 4 Department of Obstetrics & Gynaecology, The University of Melbourne, Royal Women’s Hospital, Grattan Street, Parkville, Victoria, Australia; 5 Centre for Neuroscience, The University of Melbourne, Grattan Street, Parkville, Victoria, Australia; 6 Department of Psychosis Studies, Institute of Psychiatry, King’s College London, London, UK; 7 MRC Cognition and Brain Sciences Unit, University of Cambridge, Cambridge, England, UK; 8 Melbourne School of Psychological Sciences, University of Melbourne, Grattan Street, Parkville, Victoria, Australia

**Keywords:** Preterm, Extremely low birth weight, Cogmed, Cognitive training, Working memory, Academic outcomes, Neuroplasticity, Magnetic Resonance Imaging, Randomised controlled trial

## Abstract

**Background:**

Very preterm children exhibit difficulties in working memory, a key cognitive ability vital to learning information and the development of academic skills. Previous research suggests that an adaptive working memory training intervention (Cogmed) may improve working memory and other cognitive and behavioural domains, although further randomised controlled trials employing long-term outcomes are needed, and with populations at risk for working memory deficits, such as children born preterm.

In a cohort of extremely preterm (<28 weeks’ gestation)/extremely low birthweight (<1000 g) 7-year-olds, we will assess the effectiveness of Cogmed in improving academic functioning 2 years’ post-intervention. Secondary objectives are to assess the effectiveness of Cogmed in improving working memory and attention 2 weeks’, 12 months’ and 24 months’ post-intervention, and to investigate training related neuroplasticity in working memory neural networks 2 weeks’ post-intervention.

**Methods/Design:**

This double-blind, placebo-controlled, randomised controlled trial aims to recruit 126 extremely preterm/extremely low birthweight 7-year-old children. Children attending mainstream school without major intellectual, sensory or physical impairments will be eligible. Participating children will undergo an extensive baseline cognitive assessment before being randomised to either an adaptive or placebo (non-adaptive) version of Cogmed. Cogmed is a computerised working memory training program consisting of 25 sessions completed over a 5 to 7 week period. Each training session takes approximately 35 minutes and will be completed in the child’s home. Structural, diffusion and functional Magnetic Resonance Imaging, which is optional for participants, will be completed prior to and 2 weeks following the training period. Follow-up assessments focusing on academic skills (primary outcome), working memory and attention (secondary outcomes) will be conducted at 2 weeks’, 12 months’ and 24 months’ post-intervention.

**Discussion:**

To our knowledge, this study will be the first randomised controlled trial to (a) assess the effectiveness of Cogmed in school-aged extremely preterm/extremely low birthweight children, while incorporating advanced imaging techniques to investigate neural changes associated with adaptive working memory training, and (b) employ long-term follow-up to assess the potential benefit of improved working memory on academic functioning. If effective, Cogmed would serve as a valuable, available intervention for improving developmental outcomes for this population.

**Trial registration:**

Australian New Zealand Clinical Trials Registry ACTRN12612000124831.

## Background

Approximately 15 million births per year are delivered preterm (<37 weeks’ gestation), and this number is increasing [1]. In Australia in 2010, 7.7% of liveborn infants were preterm, with 1.2% born very preterm (VPT; <32 weeks’ gestation) and 0.5% born extremely preterm (EPT; <28 weeks’ gestation) [2]. The significant advances in perinatal and neonatal medicine over the past few decades mean that the vast majority of these infants now survive to adulthood [3], so that the absolute number of preterm children in the community has increased. Preterm children, however, exhibit higher than expected rates of developmental problems [4]. It has been estimated that at least 55% of EPT children experience developmental difficulties [4], including intellectual, educational, and social-emotional impairments [4-6]. Consequently, the focus of research in preterm children has shifted from increasing survival rates to enhancing the quality of life and improving outcomes for these at-risk infants.

Working memory is a core cognitive skill critical for learning, developing basic academic skills, planning, and problem solving [7-11]. It provides the capacity for the temporary storage and manipulation of information in the course of everyday activities, such as following instructions and mental arithmetic [12-14]. A recently published meta-analysis revealed that reading-impaired children performed approximately 1 standard deviation (SD) below typically achieving students on measures of verbal working memory, and nearly ⅔ SD below peers on measures of visual working memory [7]. Mathematics-impaired children performed approximately 1 SD below typically achieving peers on verbal working memory tasks and ½ SD below on visual working memory tasks [7]. These findings support the premise that working memory capacity is critically important for academic achievement in the general population. Importantly, the relationship between working memory and academic achievement appears to be independent of general intelligence, as this relationship persists after controlling for IQ and when restricted to children with average IQs [11,12].

School-aged EPT children perform more poorly on measures of working memory and are at greater risk of exhibiting working memory deficits than their term-born peers [15-21]. As is the case in the general population, working memory has also been associated with academic performance in VPT children. Mulder and colleagues reported high correlations between verbal working memory and academic achievement in VPT children, with correlation coefficients ranging from 0.42 to 0.61 [22]. The association between working memory and academic achievement is particularly pertinent in the VPT/EPT population as it is well established that these children perform poorer on measures of basic academic skills, have elevated rates of academic underachievement and grade retention, and use more educational remediation services than term born children [4,23,24]. For example, a recent meta-analysis of 14 studies reported that VPT or very low birthweight (VLBW) children score on average 0.5 SD lower on tests of reading, 0.8 SD lower on tests of spelling, and 0.6 SD lower on tests of mathematics compared with term children [25]. These difficulties are even more prominent in children born at earlier gestational ages and with lower birthweight. It has been shown that 11-year- old EPT children score on average 1 SD below term peers on reading tests and 1.5 SD below on measures of mathematics, with 52% of EPT children classified as reading impaired and 70% as mathematically impaired [23].

The adverse social and economic consequences of academic underachievement are substantial and include low rates of post-secondary school education, high rates of unemployment, low salary in adulthood, emotional difficulties and low self-esteem [26-29]. In 1990, the economic cost of special education assistance for low birthweight children was conservatively estimated to be $371 million per annum in the USA [30], but considering inflation, the increase in the number of preterm births, and the improved survival rate of those at the edge of viability, this figure would now be much higher. In Australia, the healthcare and educational support costs for children born EPT/ELBW are substantial. Recent findings from the Longitudinal Study of Australian Children (LSAC) have indicated that community-based healthcare for mild (32–36 weeks’ gestation, birthweight 1500–2499 g and/or small for gestational age (SGA)) and moderate-to-high risk children (<32 weeks’ gestation, birthweight <1500g and/or extremely SGA) can cost, on average, an additional A$32 million per annum up to the age of 9 years compared with children with no increased perinatal risk (>36 weeks’ gestation, birthweight >2500g) [31]. Given the social and economic implications of EPT birth, it is critical that new preventative approaches are developed to improve outcomes in the preterm population.

Several cognitive training programs have been trialled to improve working memory capacity in recent years. In general, these approaches have been successful in improving an individual’s performance on specific working memory activities, but have not translated to improvements in everyday functions such as academic performance [32,33]. While repeated performance almost always results in enhanced functioning on that task, the success of cognitive training is ultimately measured by how well the training can be transferred to non-trained tasks [34]. Few cognitive training programs demonstrate transfer effects to other functions and scepticism exists regarding the functional benefits of cognitive training [35]. However, a working memory training program known as Cogmed, originally developed by Klingberg and colleagues for children with Attention Deficit Hyperactivity Disorder (ADHD) [34,36,37], has been shown to improve working memory capacity as well as lead to improvements in other untrained activities in children. Improvements in working memory capacity, as measured by performance on untrained working memory tasks post-training (near-transfer effects), have been reported in a number of studies from various populations [38-43]. These improvements in untrained working memory tasks may be attributed to the adaptive feature of Cogmed, where the demand (i.e. cognitive load) of activities is monitored and adjusted during training so that it remains close to the capacity of the child. Furthermore, compared with other known working memory programs, Cogmed does not teach explicit memory strategies, and attempts to maintain motivation by presenting activities in a fun computer game environment that is coupled with positive feedback and rewards.

Evidence supporting Cogmed was initially obtained from studies involving children with ADHD. A randomised controlled trial (RCT) of 53 children with ADHD revealed evidence of improved outcomes immediately and 3 months’ post-intervention in non-trained working memory tasks, inhibitory control, problem solving, and inattention and hyperactivity/ impulsivity symptoms compared with a placebo (non-adaptive) program (a program that included the same activities as the adaptive program but set at a lower difficulty level that does not increase progressively) [36]. These findings suggest that benefits associated with Cogmed were transferred to other aspects of cognitive functioning and everyday behaviour. Benefits of Cogmed have also been reported in other populations. In an un-blinded, non-randomised, school-based study of school-aged children assessed to have “low working memory capacity”, Holmes and colleagues [39] found that children who trained with Cogmed demonstrated long-term improvements in working memory, while children exposed to the placebo program exhibited only marginal improvements in working memory. By 6 months’ post-intervention, adaptive training was associated with an improvement in mathematical reasoning compared with pre-training baseline levels (mean difference of 0.5 SD). A more recent randomised, placebo-controlled (non-adaptive and no intervention groups) trial conducted within the school setting, replicated the benefit of adaptive training on a range of untrained working memory tasks. However, selective enhancement on academic attainment and other cognitive skills was not found either immediately after training or 1 year later [40].

The generalisability of improvements to other cognitive abilities following Cogmed training has been a topic of debate within the academic community. While a small group of studies have illustrated gains to other untrained cognitive and academic skills [36,39], reviews of the existing literature have indicated that there is insufficient research evidence to support these transfer effects [44-46]. Methodological shortcomings of previous studies likely explain these inconsistent findings, with several studies failing to employ well-validated working memory measures, an appropriate active control group, adequate sample sizes with sufficient power, and a random treatment allocation design [40,45,46]. The assessment of a benefit of improved working memory capacity on basic educational skills is likely to be a prolonged and ongoing process, with previous studies having been limited to 3- to 6-months follow-up. To overcome this limitation, a large RCT of Cogmed is currently being conducted (n=175 in each arm, 350 in total) in a community sample of Grade 1 (second year of formal primary schooling in Victoria, Australia; ages 6–7) children with “low working memory capacity” within the school setting, with follow-up at 12 months’ and 24 months’ post-intervention [47].

In the preterm population, 2 small, non-randomised, home-based studies have been published examining Cogmed [38,41]. The earlier study was conducted in a small sample of 16 extremely low birthweight (ELBW, <1000 g) and 19 term-born adolescents [41], while the latter was carried out in 20 VLBW preschoolers [38]. Consistent with other studies of Cogmed, benefits were observed in trained and untrained memory tasks in both the ELBW and term adolescents exposed to Cogmed, as well as the VLBW preschoolers. Although these studies provide initial support for Cogmed within the preterm population, the studies were observational, focused on age groups when basic academic skills are either yet to be established or largely established, had limited follow-up to 6 months' post-intervention at the latest, and did not assess academic skills. Early school-age is the ideal time to intervene to improve basic academic skills as these skills are in a critical period of development at this time.

A novel method of assessing the impact of adaptive training on working memory is to use Magnetic Resonance Imaging (MRI) to explore changes to working memory neural networks following adaptive training. Different MRI sequences are able to investigate structural and functional working memory networks. Functional MRI (fMRI) studies consistently demonstrate the importance of the dorsolateral prefrontal and the inferior parietal cortices in working memory [48-54]. Friedman and Goldman-Rakic [50] first suggested that these regions may represent important nodes of a working memory network by demonstrating concurrent metabolic activation during working memory tasks in monkeys. In children, working memory capacity has also been demonstrated to correlate with fMRI activation in the superior frontal and intraparietal regions, and activation of these regions increase with age in middle childhood as working memory capacity continues to develop [51].

Repetitive stimulation of synapses can cause neural changes that influence behaviour [55]. Such neuroplasticity can be detected by MRI. Specifically, fMRI has shown that adaptive working memory training can induce increases in activation in prefrontal and parietal regions [56,57]. Another MRI modality, diffusion weighted imaging (DWI), can detect changes to white matter connectivity and organisation, as measured by tractography and diffusion values. Changes in diffusion values such as fractional anisotropy and radial diffusivity can be attributed to growth of axonal neurofibrils such as microtubules and neurofilaments, which may occur when tasks are practiced [58]. Studies have illustrated that the maturation of white matter fibre tracts, such as the superior longitudinal fasciculus, positively correlate with the functional activation of frontal and parietal regions involved with working memory [48,59]. Furthermore, a study revealed that structural connectivity is improved in the white matter fibre tracts of the working memory system following a working memory training intervention [60]. While these findings are based on small observational studies, they provide initial evidence that the functional improvements observed through cognitive training correspond with neuroplasticity. It is plausible that the cortical maps for nodes in the working memory network may expand and their connections may strengthen as a result of intensive, sustained and adaptive training. Furthermore, because the fronto-parietal network is known to be multi-modal (i.e. involved in executive control, response inhibition, and processing of emotional stimuli), strengthening this network may partly explain the improvements seen in other domains following working memory training. Nonetheless, this has yet to be confirmed and RCTs assessing neuroplasticity following Cogmed are needed.

### Study objectives

The primary objective of this RCT is to assess the effectiveness of the Cogmed adaptive working memory training program in improving academic functioning at 24 months’ post-intervention, compared with a placebo training program, in participants recruited from a large regional cohort of EPT/ELBW 7-year-old children.

The secondary objectives are to assess the effectiveness of Cogmed in improving working memory capacity and attention at 2 weeks’, 12 months’ and 24 months’ post-intervention between the intervention and placebo groups, and to investigate training-induced neuroplasticity associated with adaptive working memory training 2 weeks following the completion of the program.

## Methods and design

### Registration and ethics approval

The trial is registered with the Australian New Zealand Clinical Trials Registry (ACTRN 12612000124831) and has been granted ethics approval by the Royal Children’s Hospital (32036), Royal Women’s Hospital (11/32), Southern Health Research Directorate (05035C), and Mercy Health (R12/37) Human Research Ethics Committees. Ethics approval has also been obtained from the Victorian Department of Education and Early Childhood Development (2012_001565) and the Catholic Education Office (GE12/0009).

### Design and setting

This study is a double-blind placebo-controlled, randomised trial. This trial will be conducted and reported according to CONSORT guidelines. Recruitment/enrolment, randomisation/start-up session, and implementation of the intervention will be carried out over an 18 month period, with participants followed up for a total of 24 months’ post-intervention. The study is therefore expected to run for approximately 42 months. The research will primarily be conducted at the Murdoch Childrens Research Institute (MCRI), Melbourne, Victoria.

### Participants

The 221 EPT/ELBW children from the 2005 Victorian Infant Collaborative Study (VICS) cohort will be invited to participate in the RCT [61,62]. The 2005 VICS cohort comprises all EPT/ELBW children born in the state of Victoria in 2005 who survived to 2 years of age. he cohort is being prospectively followed up at 7 years of age to assess school-age outcomes, which will form part of the baseline assessment for the RCT (i.e. neuropsychological assessment and certain parent questionnaires). These follow-up assessments are being carried out across the 3 study sites, the Royal Women’s Hospital (n = 95), Mercy Hospital for Women (n = 72) and Monash Medical Centre (n = 54). The participation rate achieved at the 2 year follow-up for VICS was 95%, and it is conservatively expected that 90% of the cohort will be followed up at 7 years of age. We expect approximately 60% of the VICS cohort who attend this follow-up for VICS, to be eligible for the RCT and agree to participate in the trial, and thus we aim to recruit 126 participants into the current study.

### Recruitment

Families in the 2005 VICS cohort will be invited to take part in the trial via an information card, presented to them by a research nurse coordinator during their 7 year follow-up for VICS. At this time, families will be asked if they are interested in being contacted by a researcher from the trial, who will provide them with more information about the trial.

Families not refusing contact will be contacted by the trial research assistant within 2 weeks of their 7 -year follow-up assessment to provide families with further information on the study, determine willingness to participate, and conduct a brief phone eligibility screening. The screening questionnaire will include questions regarding which school their child attends and whether their child has any sensory or developmental difficulties that may affect their ability to use a computer and/or perform computer-based activities. At this time, caregiver involvement and availability will also be discussed. Families who indicate that they do not want to participate in the trial will not be contacted further.

To participate in the trial, families must provide written consent. While there is a MRI component to the study, this is optional and children can still participate in the trial if families do not wish their children to have a MRI. Eligible and interested families will be posted the information statement and consent form and a follow-up phone call will be made to these families 1–2 weeks later. Families will be provided with a reply-paid envelope to send back the original copy of the consent form and an appointment letter with an initial study appointment date will be posted to families. A reminder phone call will be made to families prior to their first visit.

Consent from parents will also be sought to contact the child’s teacher, who will be asked to participate by completing some questionnaires relating to the child’s behaviour and school performance. Prior to contacting and seeking consent from the child’s teacher, consent from the school principal will first be sought to contact the teacher. This will be done by sending an information letter and permission slip to the school principal. If the school principal consents to the teacher participating in the study, he or she will forward the questionnaires and teacher information letter and consent form to the child’s teacher.

### Eligibility criteria

#### Inclusion criteria

Children who are part of the 2005 VICS cohort are eligible for this trial. This cohort includes all children born <28 completed weeks of gestation or with birth weight <1000 g in the state of Victoria in 2005 who survived to 2 years of age.

#### Exclusion criteria

Children with a severe intellectual, sensory or physical impairment that affects their capacity to attend mainstream school will be excluded. We expect that approximately 5% of the VICS cohort (n=11) will have an impairment that affects their capacity to attend mainstream school (such as severe intellectual impairment, moderate to severe cerebral palsy, blindness or deafness) based on the 2 year assessment. Families and/or primary caregivers who are unable to support/assist their child through to the completion of the intervention program will also be excluded. The latter will be determined by the trial research assistant and project coordinator through discussions with primary caregivers/parents during the recruitment process.

### Randomisation

Prior to the Cogmed start-up session , the child will be randomised to either the adaptive working memory training program (intervention) group or the non-adaptive working memory training program (placebo) group in a 1:1 ratio. A biostatistician who is independent of the study will generate the randomisation schedule using block randomisation with variable block sizes. Randomisation will be stratified by singleton versus multiple births (twins/triplets) and ‘low’ versus ‘age-typical’ working memory capacity at baseline (total of 4 strata), to ensure equal allocation of these factors across the 2 treatment arms. For this trial, “low” working memory capacity will be classified as performing less than or equal to the 20th percentile on the Backward Digit Recall subtest from the Working Memory Test Battery for Children (WMTB-C) [63]. Approximately 40% of participants will be classified as ‘low’ working memory, based on recently published data from a 7 year follow-up study carried out by the Victorian Infant Brain Studies (VIBeS) group [64]. Twins and triplets (approximately 23%) will be allocated to the same intervention group to reduce contamination.

Randomisation will be conducted via opaque envelopes managed by the project co-ordinator and research assistant. Participants will be allocated the next available sequential study number in the required strata and this corresponds to an envelope which will contain the allocation to intervention or placebo group. The project co-ordinator or research assistant will open the envelope to obtain the treatment allocation either on the morning of the Cogmed start-up session or the night before, depending on the time and location of the session. The participant and their family will remain blinded to the assigned treatment allocation throughout the study. Other than the project co-ordinator and research assistant, all other members of the trial team will also remain blinded to treatment allocation throughout the study.

### Intervention

Cogmed (RM version), is an adaptive working memory training program designed for children aged 7 years and up. The training is built into a series of interactive, computerised activities, which in this study are carried out in the child’s home. The program involves 25 training sessions carried out over a 5 to 7 week period. Each session runs for approximately 35 minutes and comprises 8 different working memory tasks. For the first 5 sessions, children train on the same games. On the 6^th^ session and every 5^th^ session thereafter, a new game is introduced, replacing 1 of the existing games. The program matches the complexity level of individual games with the child’s current working memory capacity on a session-by-session basis such that the complexity of the task increases adaptively with the child’s capacity.

A placebo, non-adaptive version of Cogmed was designed for the purpose of trial evaluations [36,39]. The placebo version again involves 25 sessions carried out over 5 to 7 weeks. Each session will also run for approximately 35 minutes and consists of 8 games. In this version, tasks are set to a low level of complexity throughout the training period to ensure the training does not tax working memory, but instead provides a control for the experience of sitting in front of a computer and engaging with tasks. This non-adaptive (placebo) version of the program will be administered to participants randomised to the placebo group.

The training period refers to the time from the first training session in the home to either the last training session (25^th^ training session) or the end of the 7^th^ week, depending on which occurs first. Both versions of the Cogmed training program are set-up by a Cogmed coach (the trial’s project co-ordinator or research assistant). Depending on whether the family has consented to neuroimaging and the availability and schedule of the family, the Cogmed start-up session will be arranged either at Murdoch Childrens Research Institute or at the family home. This flexible arrangement minimises the number of visits families need to make to the Murdoch Childrens Research Institute. If consent was not given for the neuroimaging aspect of the study, the Cogmed coach will visit the family home to conduct the Cogmed start-up session and set-up a laptop computer with the allocated training program (Cogmed or placebo). However, if neuroimaging consent was received, the Cogmed start-up session will be conducted at the Murdoch Childrens Research Institute on the same day as the MRI scan, and families will be given the computer to take home after the session.

During the Cogmed the start-up session, the Cogmed coach will explain how the training program works and will teach both the child and caregivers how to log into the system and complete training. The coach will also discuss planning and structuring the training with the child and their caregivers. Caregivers will also be given information on how to assist their child through the training program. Given that internet access is required for monitoring and recording the child’s progress, all families will be provided with (or reimbursed for) internet connection for the duration of the training period. Through a secure online server, the Cogmed coach will monitor the child’s training and compliance, which will be automatically captured by the training program itself. Program compliance will be assessed as the number of sessions completed by each child. The time spent per session will also be recorded. The Cogmed coach will contact families by phone each week to enquire about their progress and answer any questions from families regarding the program or the study.

### Procedures and measures

Study data will be collected via parent, teacher and self-report questionnaires, neuropsychological assessment focusing on working memory, attention and academic skills, and MRI. Table 1 outlines the study’s outcome measures and the time-points at which they will be collected.

**Table 1:** **Outcome measures and study time**-**points**

**Outcome measures**			**Time-point**^**#**^			
			Baseline*	**2 week**	**12 month**	**24 month**
**Academic achievement**	Wide Range Achievement Test-4th edition (WRAT-4)	Child	•	•	•	•
	Academic performance questionnaire	Parent	•	•	•	•
		Teacher	•			•
**Working memory**	Working Memory Test Battery for Children (WMTB-C)	Child	•	•	•	•
	Automated Working Memory Assessment (AWMA)	Child	•	•	•	•
**Attention**	Test of Everyday Attention for Children (TEACh)	Child	•	•	•	•
**Speed of processing**	CogState	Child	•	•	•	•
**General Cognitive ability**	Differential Ability Scale-2nd edition (DAS-II)	Child	•			
**Behaviour**	Strength & Difficulties Questionnaire (SDQ)	Parent	•	•	•	•
		Teacher	•			•
	Behavior Rating Inventory of Executive Function (BRIEF)	Parent	•	•	•	•
		Teacher	•			•
	Cogmed Training Evaluation Scales	Parent	•	•		
**Motivation**	Intrinsic Motivation Scale	Child	•			
	Intrinsic Motivation Inventory	Child		•		
**Neuroimaging**	MRI	Child	•	•		

^#^The presence of the ‘•’ symbol in Table 1 indicates when an outcome measure will be collected at a specific time-point from either a child or parent/teacher.

^*^Neuropsychological measures and behavioural questionnaires at the baseline time-point were completed prior to enrolment into the trial as part of a regional follow-up program.

#### Primary outcome

The primary outcome of the study is academic achievement 24 months’ post-intervention as measured using the Wide Range Achievement Test -4th edition (WRAT-4) [65]. The WRAT-4 includes subtests that assess reading (single word decoding), sentence comprehension, spelling, and math computation. Each scale is age standardised with a mean of 100 and SD of 15. Secondary information regarding literacy and numeracy, academic performance, grade repetition, and integration assistance will be sought from parents and teachers through an academic performance questionnaire.

#### Secondary outcomes

Working memory will be assessed using subtests from the Working Memory Test Battery for Children (WMTB-C) [63]. Verbal immediate memory will be assessed with the Digit Recall and Word List Recall subtests, which require the child to immediately recall verbal information. Visual-spatial immediate memory will be assessed with the Block Recall and Mazes Memory subtests whereby the child must immediately recall visual-spatial information. Verbal working memory will be assessed with the Backward Digit Recall subtest which requires the child to recall sequences of digits in the reverse order to that presented. In addition to the WMTB-C subtests, visual-spatial working memory will be assessed with Backward Block Recall, whereby the child must recall sequences of tapped blocks in the reverse order to that presented. A subtest from the Automated Working Memory Assessment (AWMA), known as Mister X, will also be used to assess visuo-spatial working memory [66].

Attention will be assessed using subtests from the Test of Everyday Attention for Children (TEACh) [67]: Sky Search (selective attention), Score! (sustained attention) and Creature Counting (shifting attention). Reaction time and decision making abilities (i.e. speed of processing) will be assessed using the Detection and Identification tasks from a computerised cognitive test battery (CogState Ltd, Melbourne, Australia). These tasks have been shown to be valid and reliable measures of speed of processing [68,69]. General cognitive ability will be assessed with the general cognitive ability score (GCA) from the Differential Ability Scale-2nd edition (DAS-II) [70]. The DAS-II is a well validated measure with excellent psychometric properties and is age standardised with a mean of 100 and SD of 15.

Behaviour will be evaluated using parent and teacher versions of the Strength & Difficulties Questionnaire (SDQ) [71]. The SDQ is a well-validated questionnaire that assesses overall behaviour problems, emotional symptoms, hyperactivity/inattention, peer relationship problems, and prosocial behaviour. Everyday manifestations of attention and working memory problems will be evaluated with the parent and teacher versions of the Behavior Rating Inventory of Executive Function (BRIEF) [72]. Parents will also be asked to complete a training evaluation scale prior to and following Cogmed training. This training evaluation is an integrated component of the Cogmed training model and seeks to gather information from parents regarding their child’s attention, hyperactivity and impulsivity.

In order to measure the child’s level of motivation and interest prior to, during, and after Cogmed training, several motivation questionnaires will be administered. Prior to commencing training, the Intrinsic Motivation Scale will be administered by the Cogmed coach to the child [73]. To assess motivation during the Cogmed training period, the Cogmed coach will ask the child a set of 7 questions concerned with how fun, motivating, and challenging the training has been for them. These questions will be asked after the first week of training, midway through the training and at the end of the training period. Following Cogmed training, a modified version of the Intrinsic Motivation Inventory (IMI) will be administered [74,75].

**Figure 1:**
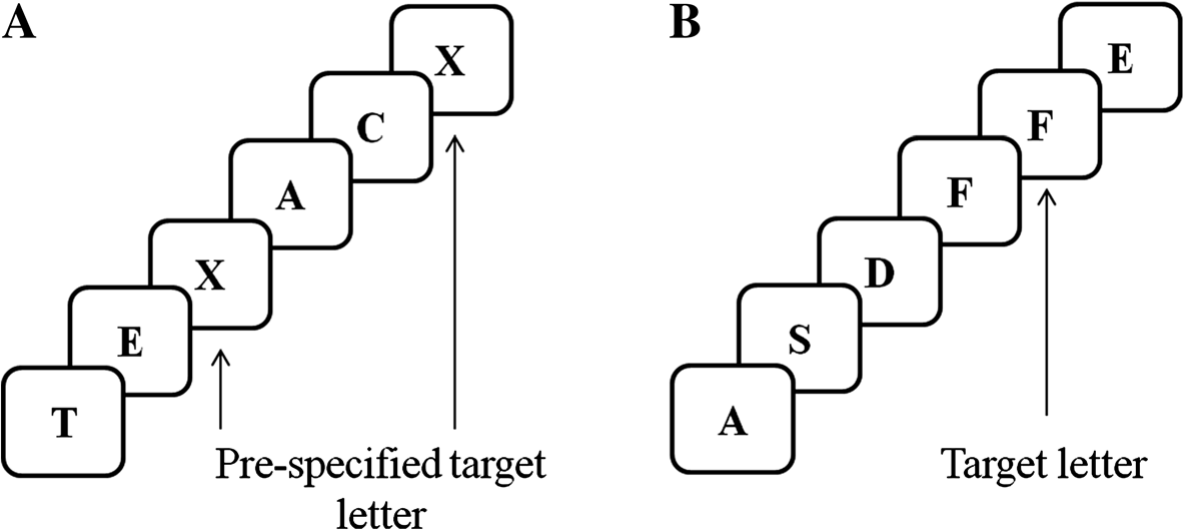
A schematic example of the A) 0-back and B) 1-back conditions of the n-back task.

Neuroimaging pre-intervention and 2 weeks’ post-intervention will be performed using a Siemens 3 Tesla TIM Trio MRI scanner located at the Royal Children’s Hospital. A) High resolution structural sequences will be performed in order to transform MRI images into a common coordinate system for further functional and diffusion image analyses. The following sequences will be acquired: a) 3D T1-weighted (MPRAGE) with high resolution 0.8 mm^3^ isotropic voxels, and b) 3D T2-weighted with 0.9mm^3^ isotropic voxels. B) Diffusion-weighted imaging (DWI) sequences will be acquired with 2.3 mm^3^ isotropic voxels with 30 gradient directions at a b value of 1000 s/mm^2^, as well as 45 gradient directions at a b value of 3000 s/mm^2^. In addition, reversed phase-encode blip images will also be collected to correct for susceptibility distortions in the spin-echo DWI images [76]. Standard diffusion metrics will be obtained by applying the diffusion tensor model to the low value DWI data (b=1000). Probabilistic tractography will be conducted using constrained spherical deconvolution on the high value DWI data (b=3000) [77]. White matter fibre tracts previously shown to relate to working memory will be identified, including the superior longitudinal fasciculus and cingulum bundle [48,59]. Tract volume and measures of axonal density (fractional anisotropy (FA) and axial diffusivity (AD)) will be extracted from within these tracts of interest. C) Functional MR echo planar images will be acquired while participant complete an established working memory task (n-back); TR=2400ms, 3.3 mm^3^ isotropic voxels. The n-back task is a well-established working memory paradigm, in which participants are asked to look at a sequence of stimuli presented and indicate for each presented stimuli, if they remember seeing it previously [78,79]. The task will comprise of 1 conditions: 1) 0-back condition and 2) 1-back condition. In the 0-back condition, children will be asked to respond if a pre-specified letter (“X”) appears on the screen, while in the 1-back condition children will respond when the letter presented on the screen is the same as the letter presented immediately before (e.g. A S D F F; Figure 1). A low-level baseline condition will also be included in which the participant simply has to look at a white cross on the screen. In total, the task will take approximately 10 minutes to complete. Similar n-back tasks have been used successfully with school-aged children in fMRI studies and reported activation of prefrontal and parietal regions associated with working memory [80,81]. In addition, resting-state fMRI data (10 minutes) will also be acquired. Participants will be asked to lie still with their eyes closed. Previous research has found that resting-state fMRI is effective for assessing large-scale brain networks in children [82,83].

Children will be carefully instructed about the imaging process prior to the MRI scan, and will have the opportunity to practice the fMRI task during a simulated ‘mock’ MRI session, which will occur prior to the scan on the same day. This preparation process will be conducted by experienced research staff and allows children to familiarise themselves with the scanner and scanning process, and raise any concerns they may have prior to the MRI scan. Furthermore, this process assists with identifying children who are unlikely to comply with the MRI procedure and is important for the acquisition of good quality MRI images without the use of sedation.

### Sample size

**Figure 2:**
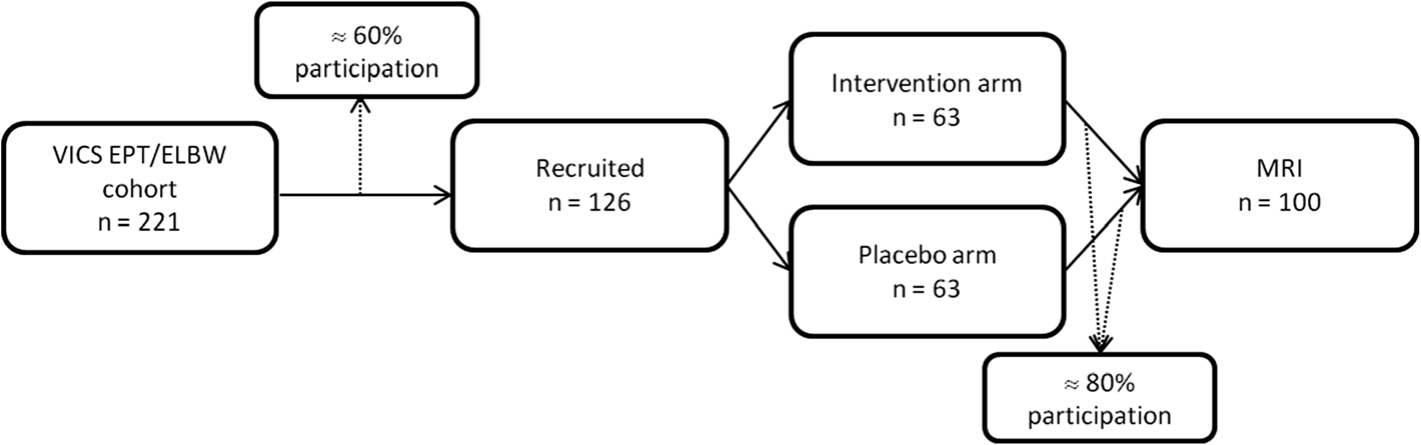
Flowchart of study participants.

All 221 children from the VICS cohort will be invited to participate in the trial providing that they fit the eligibility criteria for this study. We conservatively estimate that approximately 60% of the VICS cohort will be eligible and will consent to participate in the trial, which will provide a sample size of 126 for this study (63 per treatment group; Figure 2). A participation rate of 60% allows for children who will not be seen through the VICS follow-up (10%), as well as children who do not meet the eligibility criteria (5%; approximately n= 11). A sample size of 63 in each group will enable us to detect an increase in our primary outcome, academic functioning (such as mathematics) 24 months' post-intervention, of 0.5 SD in the intervention compared with the placebo group, with 80% power and a type-I error rate of 5%. An increase of 0.5 SD in academic outcomes is the improvement seen following Cogmed in other populations after 6 months and would be a clinically important difference to detect [39]. Of the 126 participants, we expect approximately 80% to consent to the neuroimaging component of the study, thus a sample size of 100 (50 per group) to assess the secondary objective regarding neuroplasticity. A sample size of 50 in each group with neuroimaging data will enable us to detect differences of 0.57 SD in measures of neuroplasticity (i.e. percentage BOLD signal change between pre-intervention and 2-weeks’ post-intervention for fMRI analyses, and tract volume, FA or AD for DWI analyses), with 80% power and a type-I error rate of 5%.

### Statistical analysis

All analyses will be carried out using an intention-to-treat approach, including all participants as randomised. Analysis will be carried out on those with outcome data available. Summaries of all primary and secondary outcomes will be presented by treatment group. Results will be presented as means and SDs of age standardised scores for continuous outcomes, and numbers and proportions for categorical variables. Differences between the groups on continuous outcomes will be assessed using linear regression adjusted for stratification factors of multiple births and low/normal working memory capacity at baseline. Models will be fitted using generalised estimating equations (GEEs) to allow for clustering of multiples, with results presented as the mean difference and 95% confidence intervals (CIs) calculated from sandwich estimates of standard errors. As a sensitivity analysis, results will also be adjusted for any other baseline factors where there is a chance imbalance between the 2 groups using linear regression. Differences between groups on binary outcomes will be assessed using logistic regression adjusted for stratification factors of multiple births and low/normal working memory capacity at baseline, again fitted using GEEs. Results will be presented as odds ratios (ORs) and 95% CIs calculated using sandwich estimates of standard errors. Due to the issues of multiple testing, results will be interpreted according to magnitude of the group difference rather than relying solely on significance levels. Compliance and motivation data will be presented descriptively by treatment arm.

For the fMRI data, the effects of group, time (pre-intervention and 2 weeks’ post-intervention) and task (0-back and 1-back conditions) will be examined using factorial analyses by fitting an analysis of variance (ANOVA) model [81]. fMRI effective connectivity will be examined using Dynamic Causal Modelling [82].

### Consent

Written informed consent was obtained from the participant’s parent or guardian for the publication of this manuscript and any accompanying images.

## Discussion

As the number of EPT/ELBW survivors has increased, the focus of research into preterm birth has shifted to improving long-term outcome of these children. Because EPT/ELBW children are at a greater risk of poor educational, behavioural and social outcomes than their term-born counterparts, implementation of evidence-based interventions are crucial for reducing the risk of long-term impairment in these children. Early academic underachievement can hinder later life success and job attainment, which results in additional economic and societal burden. Working memory deficits have been associated with poor school performance, both of which are exhibited by preterm children [17,22,23,25]. Emerging evidence suggests that a training intervention in the early school period can improve working memory and may in time enhance academic outcomes in term-born children. A priority now is to establish whether this potentially promising intervention can lead either to immediate impacts on working memory capacity or, more importantly, to long-term improvements in important functional outcomes such as academic skills in preterm children.

The proposed study will be the first study to investigate the effectiveness of Cogmed, an adaptive working memory training program, in the extremely preterm population within a controlled environment and assess whether improved working memory generalises to improving academic outcomes 24 months’ post-intervention. Furthermore, the incorporation of a range of advanced MRI techniques in this RCT of Cogmed is novel, and will be essential for determining and monitoring the neural changes that occur as a result of adaptive working memory training.

If effective, the Cogmed program could potentially serve as a valuable intervention for improving educational outcomes in EPT/ELBW children. In summary, this trial has the potential to provide an evidence base for a promising, new intervention for improving academic and developmental outcomes in EPT/ELBW children and will contribute to the existing literature surrounding the efficacy of Cogmed.

## Abbreviations

ADHD: Attention- deficit hyperactivity disorder; AD: Axial diffusivity; AWMA: Automated Working Memory Assessment; BRIEF: Behavior Rating Inventory of Executive Function; CI: Confidence interval; DAS-II: Differential Ability Scale-2nd edition; DWI: Diffusion-Weighted Imaging; ELBW: Extremely low birthweight; EPT: Extremely preterm; FA: Fractional anisotropy; fMRI: Functional Magnetic Resonance Imaging; GCA: General cognitive ability; GEE: Generalised estimating equations; MCRI: Murdoch Childrens Research Institute; MRI: Magnetic Resonance Imaging; OR: Odds ratio; RCT: Randomised controlled trial; SDQ: Strength & Difficulties Questionnaire; SD: Standard deviation; SGA: Small for gestational age; TEACh: Test of Everyday Attention for Children; VIBeS: Victorian Infant Brain Studies; VICS: Victorian Infant Collaborative Study; VLBW: Very low birthweight; VPT: Very preterm; WMTB-C: Working Memory Test Battery for Children; WRAT-4: Wide Range Achievement Test -4th edition.

## Competing interests

All authors declare that they have no competing.

## Authors’ contributions

PJA, LP, GR, LWD, KJL, DKT, MLS, EKJ, CN, SG have made substantial contributions to the conception and design of the study. All authors have reviewed and contributed to the final version of the manuscript. All authors have read and approved the final manuscript.

## Pre-publication history

The pre-publication history for this paper can be accessed here:

http://www.biomedcentral.com/1471-2431/13/144/prepub
